# Loss of *Paip1* causes translation reduction and induces apoptotic cell death through ISR activation and *Xrp1*

**DOI:** 10.1038/s41420-023-01587-8

**Published:** 2023-08-05

**Authors:** Maoguang Xue, Fei Cong, Wanling Zheng, Ruoqing Xu, Xiaoyu Liu, Hongcun Bao, Ying Ying Sung, Yongmei Xi, Feng He, Jun Ma, Xiaohang Yang, Wanzhong Ge

**Affiliations:** 1 Division of Human Reproduction and Developmental Genetics, Women’s Hospital, Zhejiang University School of Medicine, Hangzhou, Zhejiang 310058 China; 2 Institute of Genetics, Zhejiang University School of Medicine, Hangzhou, Zhejiang 310058 China; 3 Institute of Molecular and Cell Biology, Agency for Science, Technology and Research (A∗STAR), 61 Biopolis Drive, Proteos, Singapore, 138673 Singapore; 4 Zhejiang Provincial Key Laboratory of Precision Diagnosis and Therapy for Major Gynecological Diseases, Women’s Hospital, Zhejiang University School of Medicine, Hangzhou, Zhejiang 310006 China; 5 Cancer Center, Zhejiang University, Hangzhou, Zhejiang 310058 China

**Keywords:** Cell death, Cell signalling

## Abstract

Regulation of protein translation initiation is tightly associated with cell growth and survival. Here, we identify *Paip1*, the *Drosophila* homolog of the translation initiation factor *PAIP1*, and analyze its role during development. Through genetic analysis, we find that loss of *Paip1* causes reduced protein translation and pupal lethality. Furthermore, tissue specific knockdown of *Paip1* results in apoptotic cell death in the wing imaginal disc. *Paip1* depletion leads to increased proteotoxic stress and activation of the integrated stress response (ISR) pathway. Mechanistically, we show that loss of *Paip1* promotes phosphorylation of eIF2α via the kinase PERK, leading to apoptotic cell death. Moreover, *Paip1* depletion upregulates the transcription factor gene *Xrp1*, which contributes to apoptotic cell death and eIF2α phosphorylation. We further show that loss of *Paip1* leads to an increase in *Xrp1* translation mediated by its 5’UTR. These findings uncover a novel mechanism that links translation impairment to tissue homeostasis and establish a role of ISR activation and *Xrp1* in promoting cell death.

## Introduction

Translational control plays an important role in animal development and tissue homeostasis [1]. Dysregulation of translation is associated with various human diseases, including neurodegeneration and cancer [2–4]. One critical step of translational regulation is the initiation, which is considered as the most complex and rate-limiting step. Altered levels or activities of translation initiation factors have been reported in various human cancers [5–8]. PAIP1 is a poly (A)-binding protein (PABP) interacting protein, which functions to stimulate translation initiation through its interaction with eIF3G and eIF4A [9, 10]. It has been demonstrated that PAIP1 increases the stimulatory effect of PABP in translation initiation and promotes mRNA circulation in mammalian cells [11]. Upregulation of PAIP1 levels are reported in several types of human malignant tumors, including breast cancer, lung adenocarcinoma, and gastric cancer [12–15]. Interestingly, recurrent mutations in *PAIP1* have also been found in urothelial bladder carcinoma patients, suggesting a complex role of *PAIP1* during tumorigenesis [16]. A recent study in mice spermatogenesis suggests that mouse PAIP1 functions to promote mRNA translation through binding to YBX2 [17]. While much is known about the biochemical roles of Paip1 in translation initiation, our current knowledge of its functions and regulatory mechanisms in animal development and tissue homeostasis remains limited.

Here, we describe studies using *Drosophila* to evaluate the role of *Paip1* during development. Loss of function experiments demonstrate that *Paip1* is essential for *Drosophila* development and functions in translation control. Tissue-specific knockdown reveals that *Paip1* depletion causes tissue damage, leading to apoptotic cell death in the wing imaginal disc. Further analysis reveals that knockdown of *Paip1* induces proteotoxic stress and activates the integrated stress response pathway (ISR). We provide evidence that PERK-mediated phosphorylation of eIF2α contributes to the apoptotic cell death phenotype in *Paip1* deficient tissues. Moreover, we find that the transcription factor gene *Xrp1* is induced by *Paip1* depletion and facilitates apoptotic cell death and eIF2α phosphorylation. Further analysis reveals that loss of *Paip1* results in an increase in *Xrp1* translation through its 5’UTR. In conclusion, our data together suggest that loss of *Drosophila Paip1* causes translation reduction and induces apoptotic cell death through ISR activation and *Xrp1*.

## Results

### Loss of Paip1 causes lethality and reduces translation in Drosophila

**Fig. 1:**
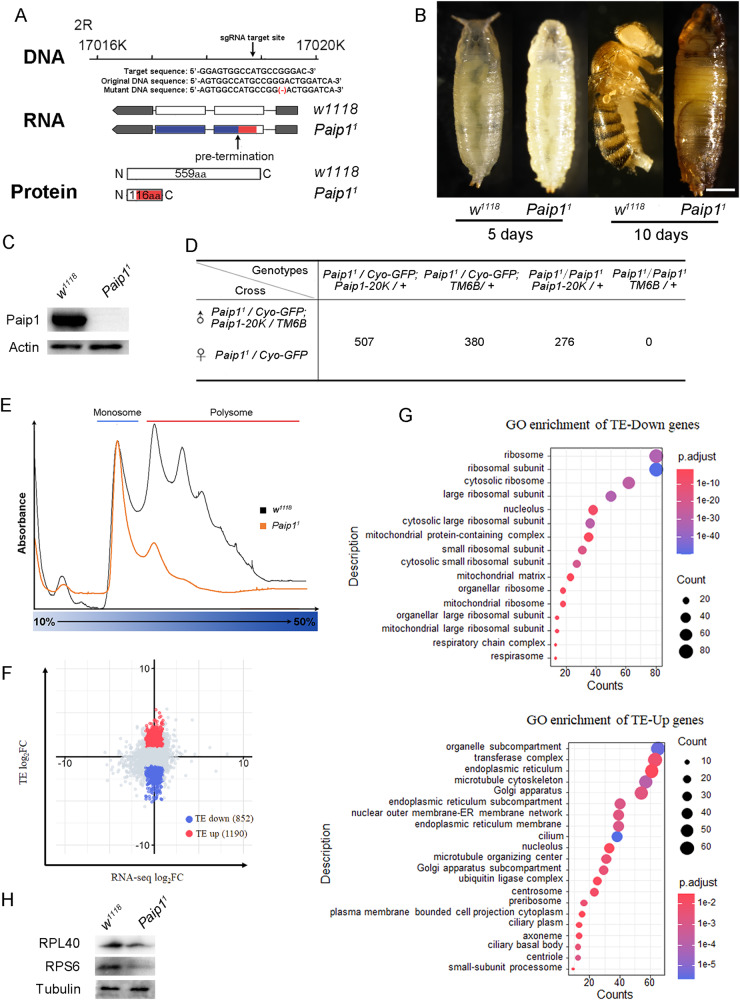
Loss of *Paip1* causes lethality and reduces translation. **A** Generation of mutation in *Drosophila Paip1*. Diagrams showing the genomic region around the *Paip1* locus, the target sequence of sgRNA, and the DNA/RNA/protein after Cas9/sgRNA induced mutation. *Paip1* mutant allele contains 1-bp deletion around the target region, which induces a codon shift and generates a truncated protein due to premature translation termination. **B** Images of pupae and adult flies with the indicated genotype. Wild-type and *Paip1* mutant pupae and adult flies were collected 5 days after egg hatching and 10 days after egg hatching. Scale bars, 500 µm. **C** Western blot of wild-type and *Paip1* mutant larvae extracts probed with anti-Paip1 and anti-Actin antibodies. The anti-Paip1 antibody detects a single 65 KD band in wild-type larvae, which was absent in *Paip1* mutants. **D** Quantification of rescue of lethality in various genotypes. Flies with the indicated genotypes were crossed. The number of progenies with the indicated genotypes was counted. A *Paip1-20K* transgene fly carries a 20-kb duplicated genomic fragment containing the entire *Paip1* gene region (CH322-46004). Introducing one copy of Paip1-20 K transgene into the *Paip1* mutant background was able to rescue the lethality defect. **E** Polysome profiles from wild-type and *Paip1* mutant larvae. Monosome fraction and Polysome fraction were collected for Polysome sequencing. **F** Scatter plot of TE log2 fold-changes to total mRNA log2 fold-changes in wild-type and *Paip1* mutant larvae with RNA-seq. The number of mRNAs with a change in TE (blue and red) are indicated (|RNA-seq log2FC| < 1 and |TE log2FC| > 1). TE, translational efficiency. Each dataset includes 2 replicates. **G** GO enrichment analysis of TE-Down genes and TE-Up genes. **H** Western blot of *w*^*1118*^ and *Paip1* mutant larvae extracts probed with anti-RPL40, anti-RPS6, and anti-Tubulin antibodies.

The *Drosophila* genome contains one single homolog of *PAIP1*, CG8963, with a DIOPT (DRSC integrative ortholog prediction tool) score of 11/15. We thereafter named *Drosophila* CG8963 as *Paip1*. *Drosophila Paip1* and Human *PAIP1* share 39% similarity and 23% identity at the amino acid level. To explore the function of *Paip1* during *Drosophila* development, we used the CRISPR/Cas9 genome editing system to generate one mutant allele, *Paip1*^*1*^ (Fig. 1A) [18]. *Paip1*^*1*^ contained a single nucleotide deletion in the *Paip1* coding sequence, leading to a frameshift mutation and induction of a premature stop codon at amino acid 117 of the Paip1 protein (Fig. 1A). The homozygous *Paip1*^*1*^ mutants were embryonic viable, and the newly hatched larvae survived to the third instar larval stage (Table S1). The *Paip1*^*1*^ mutant larvae continued to develop into pupae with a developmental delay and died during the pupal stage (Fig. 1B, Table S1), indicating *Paip1* is essential for *Drosophila* development. An antibody against Paip1 was raised, and our western blot analysis confirmed that Paip1 was absent in the *Paip1*^*1*^ mutant (Fig. 1C). For the *Paip1*^*1*^ homozygous mutant, the lethality phenotype was rescued when one copy of genomic DNA fragment containing *Paip1* was introduced into the mutant background, confirming the mutant lethality is due to the specific loss of Paip1 (Fig. 1D).

As it has been proposed that mammalian PAIP1 functions to stimulate translation in part through binding to PABP and facilitating mRNA circularization [11], we performed experiments to investigate how Paip1 controls translation in a developmental context. Translation activity of mRNAs can be monitored by polysome profiling [19]. To examine changes in the translation activities of mRNAs, wild-type and *Paip1*^*1*^ mutant larval extracts were subjected to polysome profiling (Fig. 1E). Polysomes are large complexes of translating ribosomes engaged with mRNA, and can be segregated through a sucrose gradient ultracentrifugation. RNA position in the gradient is detected by the absorbance of ultraviolet light, which produces a distinct polysome profile that gives a snapshot of global translational activity. We found that the abundance of mRNAs engaged with polysomes was reduced in *Paip1* mutants, as compared to the control, suggesting that *Paip1* is required for promoting translation in *Drosophila* (Fig. 1E).

To investigate gene-specific functions of *Paip1*, we analyzed mRNAs that were preferentially associated with monosome or polysome fractions (Fig. 1E). Here, we separated these two fractions and extracted the associated RNAs for RNA-seq (Polysome-Seq) [20]. Prior to library construction, we added a predetermined amount of ERCC spike-in reagent to each RNA samples as a control for subsequent quantification [21]. The total RNA of the corresponding genotype was extracted from larvae for RNA-seq as transcriptome input. We identified 1190 genes with upregulated translation efficiency (Polysome/monosome) and 852 genes with downregulated translation efficiency in *Paip1*^*1*^ mutant compared to the control (Fig. 1F). Gene ontology (GO) analysis revealed that the translation reduction genes included those related to the ribosome, ribosomal subunit, cytosolic ribosome, large ribosomal subunit, cilium, cytosolic large ribosomal subunit and cytosolic small ribosomal subunit (Fig. 1G). GO terms organelle subcompartment, transferase complex, endoplasmic reticulum, microtubule cytoskeleton and Golgi apparatus were enriched in the translation upregulation genes (Fig. 1G).

Based on the decrease in the translation efficiency of various ribosomal protein mRNAs, we further performed Western blot analysis to verify this effect. Our data showed that two chosen ribosomal proteins, RPL40 and RPS6, exhibited reduced protein levels in *Paip1*^*1*^ mutant compared to the control (Fig. 1H, right, quantified in supplementary Fig. S1). Taken together, these data suggest that *Paip1* is required for *Drosophila* development and has a functional role in translation control.

### Paip1 depletion results in apoptotic cell death in the wing imaginal disc

**Fig. 2:**
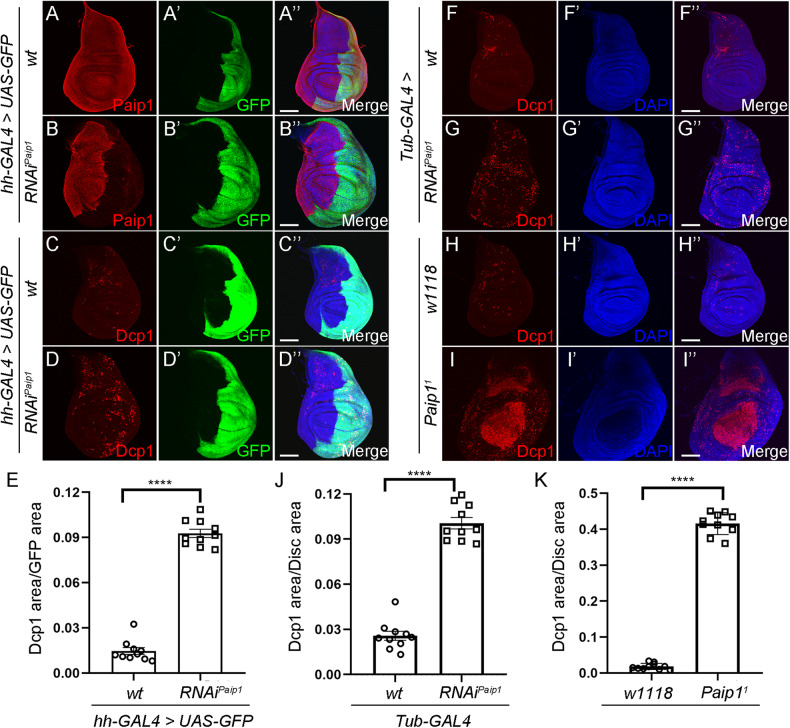
*Paip1* depletion results in apoptotic cell death in the wing imaginal disc. **A**, **B**” Validation of the efficiency of RNAi-mediated *Paip1* knockdown under *hedgehog-GAL4* (*hh-GAL4*). Wing imaginal discs from third instar larvae of control (**A**–**A**”) and *Paip1-RNAi* (**B**–**B**”) stained for Paip1 (red), GFP (green), and DAPI (blue). Knockdown of *Paip1* leads to a strong reduction of Paip1 protein level in the posterior compartment of wing imaginal disc (labeled with GFP). **C**, **D**” Knockdown of *Paip1* in the posterior compartment of wing discs induces apoptotic cell death. Wing imaginal discs from third instar larvae of control (**C**–**C**”) and *Paip1-RNAi* (**D**–**D**”) stained for Dcp1 (red), GFP (green), and DAPI (blue). The posterior compartment (labeled with GFP) in the disc of *Paip1-RNAi* shows more Dcp1 signals compared to the anterior compartment. (**E**) Statistical data of apoptotic cell death (Dcp1 area/GFP area) in (**C**), (**D**)”. **F**–**G**” Whole-body knockdown of *Paip1* under *tubulin-GAL4* (*Tub-GAL4*) induces apoptotic cell death. Wing imaginal discs from third instar larvae of control (**F**–**F**”) and *Paip1-RNAi* (**G**–**G**”) stained for Dcp1 (red) and DAPI (blue). Increased Dcp1 signals are present in *Paip1-RNAi* wing discs. **H**, **I**” *Paip1* mutation induces apoptotic cell death. Wing imaginal discs from third instar larvae of wild-type (**F**–**F**”) and *Paip1* mutant (**G**–**G**”) stained for Dcp1 (red) and DAPI (blue). Increased Dcp1 signals are present in *Paip1* mutant wing discs. **J** Statistical data of apoptotic cell death (Dcp1 area/Disc area) in (**F**–**G**)”. **K** Statistical data of apoptotic cell death (Dcp1 area/Disc area) in (**H**–**I**”). For (**A**–**D**”) and (**F**–**I**”), scale bars, 100 µm. For (**E**), (**J**), (**K**), data are mean ± SEM. *n* = 10 discs per genotype. Statistical analysis was performed using a two-tailed unpaired *t*-test. ****P < 0.0001.

*Drosophila* wing imaginal discs have been widely used to study tissue homeostasis, especially cell proliferation, differentiation, and cell death [22, 23]. To investigate *Paip1* function in a tissue-specific context, we depleted *Paip1* in the posterior compartment of the developing wing imaginal discs. Here we expressed *Paip1-RNAi* under the control of the *hedgehog (hh)-Gal4* driver. A strong downregulation of Paip1 protein levels was observed in the wing posterior compartment, indicating that *Paip1-RNAi* effectively downregulated the Paip1 levels (Fig. 2A, B’’). Previous studies have shown that loss of several translational regulators causes apoptotic cell death in the *Drosophila* wing imaginal discs [24, 25]. To evaluate the effect of *Paip1* depletion on cell death, we performed immunofluorescence staining with the anti-Cleaved Dcp-1 antibody, which labels apoptotic cells [26]. The knockdown of *Paip1* in the wing posterior compartment resulted in a strong increase of cleaved Dcp-1 positive dying cells compared to the control anterior compartment (Fig. 2C–D’’, and quantified in E). Increased cleaved Dcp-1 signals were also observed when *Paip1* was depleted in the entire wing imaginal discs by *tubulin (tub)-Gal4* driven *Paip1-RNAi* (Fig. 2F, G’’, and quantified in J). We noticed that knockdown of *Paip1* by *tub-Gal4* resulted in pupal lethality similar to the *Paip1*^*1*^ mutant (Supplementary Fig. S2). To further confirm these results, we also analyzed the anti-cleaved Dcp-1 staining intensity in *Paip1*^*1*^ mutant discs. Consistent with the RNAi experiment, *Paip1*^*1*^ mutant wing discs displayed upregulation of cleaved Dcp-1 signals (Fig. 2H, I’’, and quantified in K). Moreover, overexpression of the baculovirus effector caspase inhibitor protein p35 suppressed apoptotic cell death in *Paip1* knockdown wing discs, indicating that the cell death is caspase-dependent (Supplementary Fig. S3). Therefore, *Paip1* deficient cells undergo apoptotic cell death.

### Increased proteotoxic stress and activation of ISR in Paip1 deficient cells

**Fig. 3:**
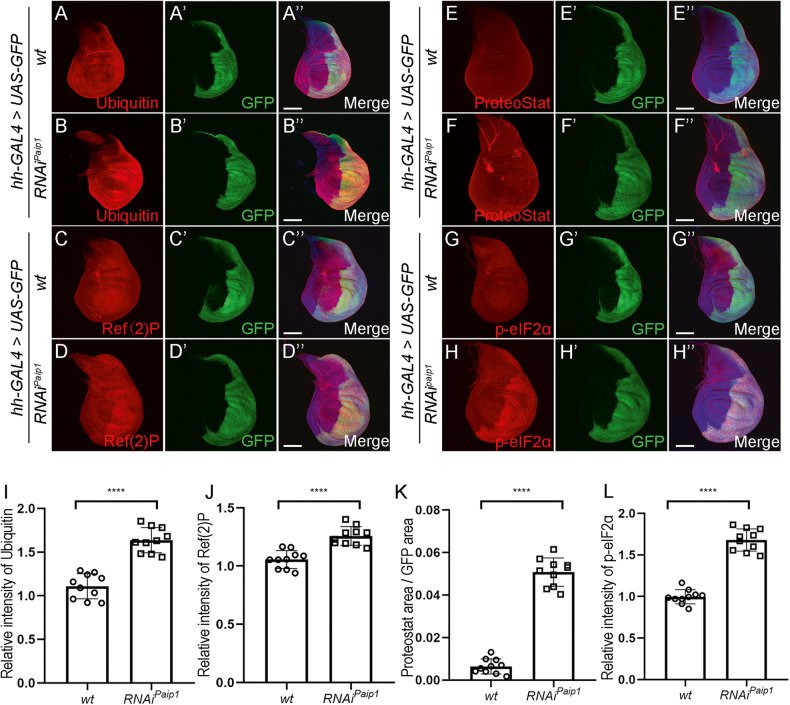
*Paip1* depletion induces increased proteotoxic stress and activation of the integrated stress response pathway. **A**, **B**” Wing imaginal discs from third instar larvae of control (**A**–**A**”) and *Paip1-RNAi* (**B**–**B**”) stained for Ubiquitin (red), GFP (green), and DAPI (blue). **C**, **D**” Wing imaginal discs from third instar larvae of control (**C**–**C**”) and *Paip1-RNAi* (**D**–**D**”) stained for Ref(2) P (red), GFP (green), and DAPI (blue). **E**, **F**” Wing imaginal discs from third instar larvae of control (**E**–**E**”) and *Paip1-RNAi* (**F**–**F**”) stained for ProteoStat (red), GFP (green), and DAPI (blue). **G**, **H**” Wing imaginal discs from third instar larvae of control (**G**–**G**”) and *Paip1-RNAi* (**H**–**H**”) stained for p-eIF2α (red), GFP (green) and DAPI (blue). **I** Statistical data of Ubiquitin level in (**A**–**B**”). **J** Statistical data of Ref(2) P level in (**C**–**D**”). **K** Statistical data of ProteoStat (ProteoStat area/Disc area) in (**E**–**F**”). **L** Statistical data of p-eIF2α level in (**G**–**H**”). For (**A**–**H**”), scale bars, 100 µm. For (**I**–**L**), data are mean ± SEM. *n* = 10 discs per genotype. Statistical analysis was performed using a two-tailed unpaired *t*-test. ****P < 0.0001.

It has been shown previously that mutations of ribosomal proteins cause widespread apoptosis and proteotoxic stress [27–29]. The elevated apoptosis in *Paip1* deficient cells suggests that these cells are likely stressed. We next tested whether depletion of *Paip1* creates proteotoxic stress in the wing imaginal discs. In order to do this, we performed immunofluorescence staining using an anti-Ubiquitin antibody, which marks the ubiquitination, one marker for proteotoxic stress [30]. Higher ubiquitination levels were detected in the wing posterior compartment by *hh-Gal4* driven *Paip1-RNAi* as compared to the anterior compartment (Fig. 3A, B’’, quantified in I). Consistently, accumulation of Ref(2)P, which marks ubiquitinated protein bodies, was observed in *Paip1* knockdown cells (Fig. 3C, D’’, quantified in J) [31]. Increased ubiquitination levels indicate abnormal protein degradation. It is likely that protein aggregates are formed due to less efficient protein degradation in *Paip1* deficient cells. We then used ProteoStat, a dye that binds selectively to aggregated proteins [32], to test this possibility. Indeed, ProteoStat staining assay revealed that depletion of *Paip1* led to accumulation of protein aggregates in the wing posterior compartment (Fig. 3E, F’’, quantified in K). Elevated proteotoxic stress is tightly associated with the activation of integrated stress response [28, 33]. To examine whether the ISR is activated in *Paip1*-deficient cells, we performed immunofluorescence staining with antibodies against phosphorylated eIF2α, which serves as a marker for ISR activation. The p-eIF2α signals were higher in the wing posterior compartment than in the control anterior compartment of the discs upon *Paip1* depletion by *hh-Gal4* driven *Paip1-RNAi* (Fig. 3G, H’’, quantified in L). The elevated p-eIF2α levels were also verified in *Paip1*^*1*^ mutant wing discs (Supplementary Fig. S4). Together, these findings suggest that depletion of *Paip1* causes increased proteotoxic stress and activates the ISR.

### Activation of ISR drives apoptosis by PERK-mediated phosphorylation of eIF2α

**Fig. 4:**
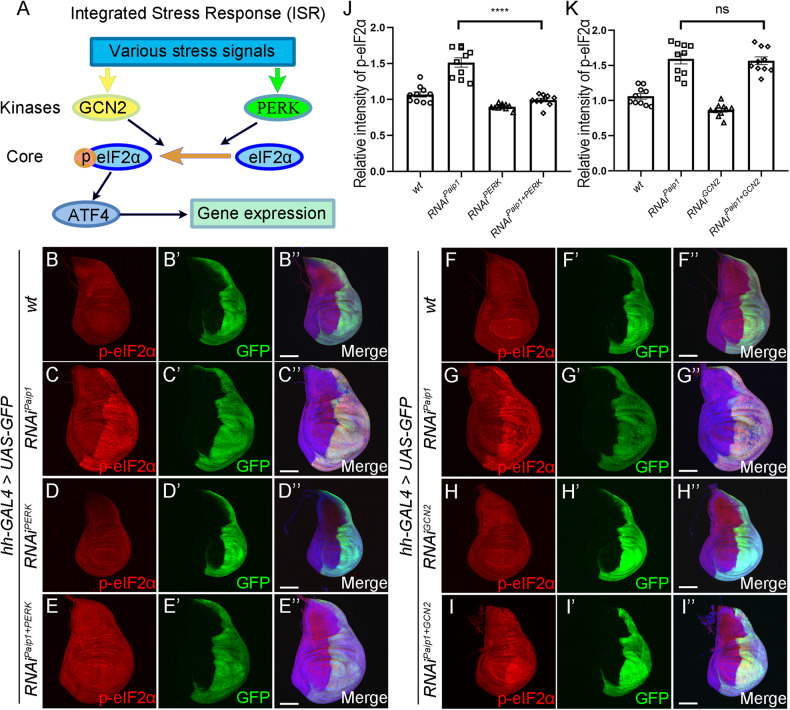
Activation of ISR is mediated by PERK. **A** Schematic of the *Drosophila* integrated stress response (ISR) pathway. Kinases (GCN2, PERK) which phosphorylate eIF2α are activated under certain stress signals. Phosphorylated eIF2α leads to translational induction of ATF4, which mediates expression of stress-related genes. **B**–**E**” PERK mediates phosphorylation of eIF2α in wing discs expressing *Paip1-RNAi*. Wing imaginal discs from third instar larvae of control (**B**–**B**”), *Paip1-RNAi* (**C**–**C**”), *PERK-RNAi* (**D**–**D**”) and *Paip1-PERK double RNAi* (**E**–**E**”) stained for eIF2α (red), GFP (green) and DAPI (blue). Higher level of phosphorylated eIF2α upon *Paip1* knockdown (**C**–**C**”, labeled with GFP) was reduced by knocking down *PERK* simultaneously (**E**–**E**”). **F**–**I**” GCN2 shows little effects on phosphorylation of eIF2α in wing discs expressing *Paip1-RNAi*. Wing imaginal discs from third instar larvae of control (**F**–**F**”), *Paip1-RNAi* (**G**–**G**”), *GCN2-RNAi* (**H**–**H**”), and *Paip1-GCN2 double RNAi* (**I**–**I**”) stained for eIF2α (red), GFP (green) and DAPI (blue). Higher level of phosphorylated eIF2α induced by *Paip1* knockdown (**C**–**C**”, labeled with GFP) was not altered by *GCN2-RNAi* (**E**–**E**”). **J** Statistical data of p-eIF2α level in (**B**–**E**”). **K** Statistical data of p-eIF2α level in (**F**–**I**”). For (**B**–**I**”), scale bars, 100 µm. For (**J**), (**K**), data are mean ± SEM. *n* = 10 discs per genotype. Statistical analysis was performed using a two-tailed unpaired *t*-test. ****P < 0.0001; ns, *P* > 0.05.

**Fig. 5:**
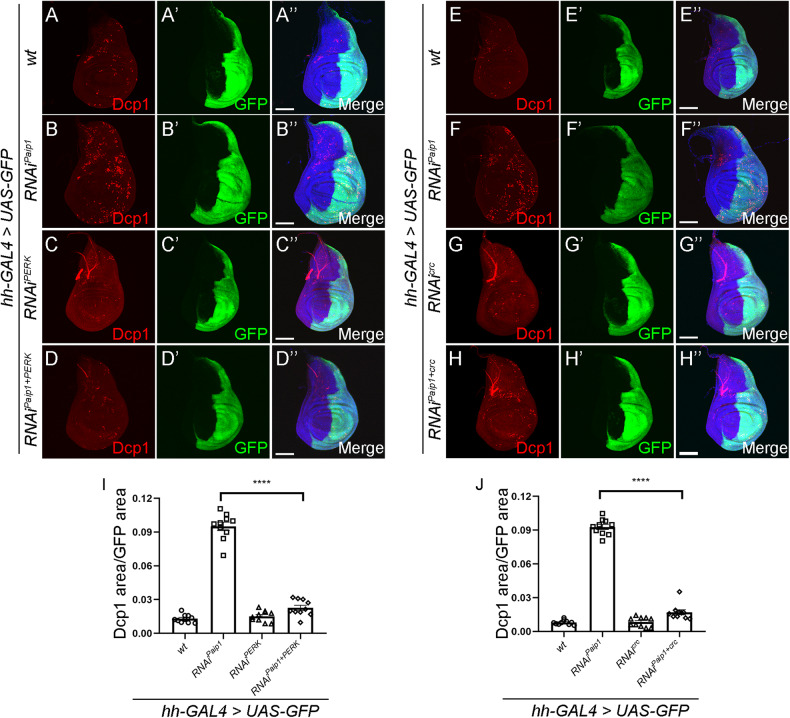
Knockdown of *PERK* or *crc* (*ATF4*) rescues apoptotic cell death induced by *Paip1* depletion. **A**–**D**” Knockdown of *PERK* suppresses cell death induced by *Paip1* depletion. Wing imaginal discs from third instar larvae of control (**A**–**A**”), *Paip1-RNAi* (**B**–**B**”), *PERK-RNAi* (**C**–**C**”), and *Paip1-PERK double RNAi* (**D**–**D**”) stained for Dcp1 (red), GFP (green) and DAPI (blue). **E**–**H**” Knockdown of *crc (ATF4)* suppresses cell death induced by *Paip1* depletion. Wing imaginal discs from third instar larvae of control (**E**–**E**”), *Paip1-RNAi* (**F**–**F**”), *crc-RNAi* (**G**–**G**”) and *Paip1-crc double RNAi* (**H**–**H**”) stained for Dcp1 (red), GFP (green) and DAPI (blue). **I** Statistical data of apoptotic cell death (Dcp1 area/GFP area) in (**A**–**D**”). **J** Statistical data of apoptotic cell death (Dcp1 area/GFP area) in (**E**–**H**”). For (**A**–**H**”), scale bars, 100 µm. For (**I**) and (**J**), data are mean ± SEM. *n* = 10 discs per genotype. Statistical analysis was performed using a two-tailed unpaired *t*-test. ****P < 0.0001.

The ISR is activated as an adaptive pathway to restore cellular homeostasis in response to various stress stimuli [34]. Meanwhile, activation of ISR in cells exposed to chronic or severe stress can also drive cell death [35]. We next tested whether activation of ISR contributes to apoptotic cell death in *Paip1* deficient cells. In *Drosophila*, the ISR is activated by two discrete kinases, PERK and GCN2 [36], that phosphorylate eIF2α to limit the availability of initiator methionyl-tRNA and suppress cap-dependent translation while allowing translation of selected genes including *ATF4* (Fig. 4A). To examine which one of the two kinases mediates the effects of *Paip1* depletion on the ISR, we knocked down individually *PERK* and *GCN2* in a *Paip1* depletion background using *hh-Gal4*. Previous reported RNAi lines for *PERK* and *GCN2* were used for this analysis [37–39]. We found that heightened eIF2α phosphorylation in *Paip1*-deficient cells was brought to normal levels by *PERK* knockdown (Fig. 4B–E”, quantified in J). Knockdown of *GCN2* failed to lead to such an effect (Fig. 4F–I”, quantified in K). To rule out the possible effect of reduced RNAi efficiency due to GAL4 dilution, we performed a control experiment to examine the rescue effect by expressing an additional *UAS-lacZ* transgene in *Paip1* knockdown background. Our results showed that the eIF2α phosphorylation levels were not altered (Supplementary Fig. S5). Furthermore, knockdown of *PERK* largely rescued the apoptotic cell death in *Paip1* depletion cells (Fig. 5A–D”, and quantified in I). Considering that ATF4 is a downstream core factor of ISR, we also knocked down *crc* (*Drosophila* homolog of *ATF4*) in the context of *Paip1-RNAi*. The results showed that *crc* knockdown could also effectively inhibit apoptosis (Fig. 5E–H”, and quantified in J). Similar to our previous control experiments, we overexpressed an additional *UAS-lacZ* transgene in *Paip1* knockdown background and found that no rescue effects for apoptosis were observed (Supplementary Fig. S6). These results indicate that activation of ISR triggers apoptosis by PERK-mediated phosphorylation of eIF2α in *Paip1* deficient cells.

### Loss of Paip1 upregulates Xrp1, which causes phosphorylation of eIF2α and apoptosis

**Fig. 6:**
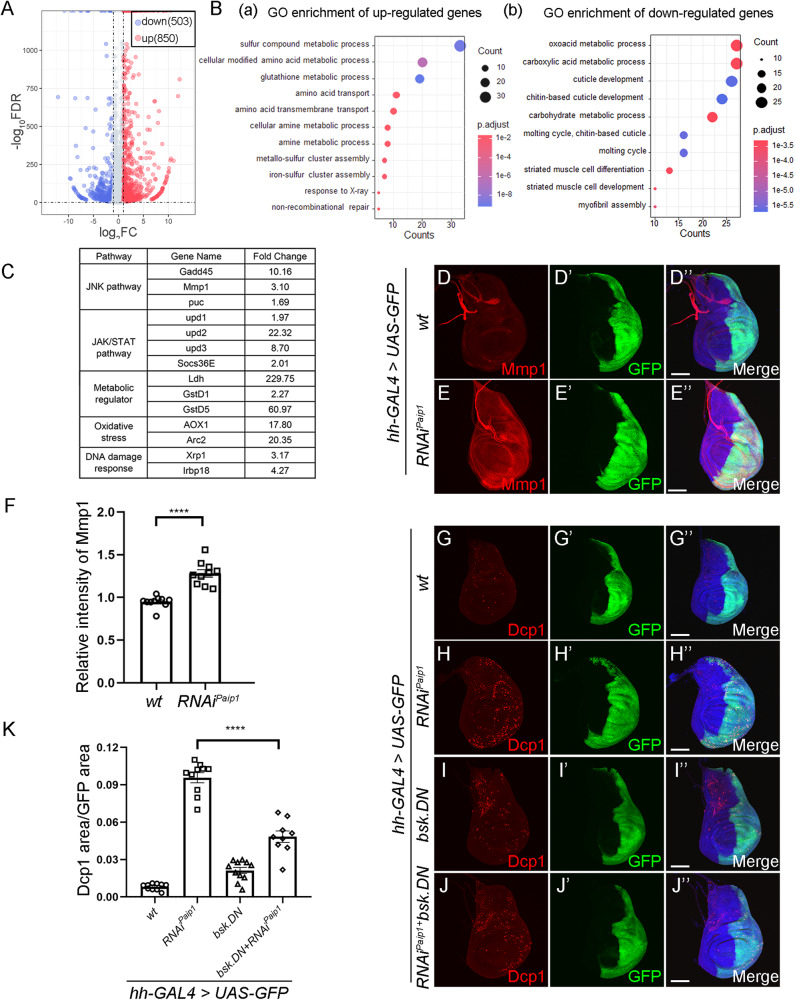
Suppression of the JNK pathway partially rescues apoptotic cell death in *Paip1* depletion wing discs. **A** Volcano plots of differentially expressed genes between control wing discs and wing discs expressing *Paip1-RNAi* under *Tub-GAL4*. The numbers of differentially expressed gene are indicated (FDR < 0.05 and |log2FC| > 1), in which blue and red denote down- and upregulated genes, respectively. Each genotype includes 2 replicates. **B** GO enrichment analysis for upregulated (a) and downregulated genes (b). The significant level is 0.05. **C** Fold changes of representative genes for selected signaling pathway through manual annotation. **D**, **E**” Wing imaginal discs from third instar larvae of control (**D**–**D**”) and *Paip1-RNAi* (**E**–**E**”), stained for Mmp1 (red), GFP (green), and DAPI (blue). **F** Statistical data of Mmp1 level in (**D**–**E**”). **G**–**J**” Wing imaginal discs from third instar larvae of control (**G**–**G**”), *Paip1-RNAi* (**H**–**H**”), *UAS-bsk.DN* (**I**–**I**”) and *UAS-bsk.DN* +*Paip1-RNAi* (**J**–**J**”) stained for Dcp1 (red), GFP(green) and DAPI(blue). **K** Statistical data of apoptotic cell death (Dcp1 area/GFP area) in (**G**–**J**”). For (**D**–**E**”) and (**G**–**J**”), scale bars, 100 µm. For (**F**) and (**K**), data are mean ± SEM. *n* = 10 discs per genotype. Statistical analysis was performed using a two-tailed unpaired *t*-test. ****P < 0.0001.

Translation defects can lead to the integrated stress response that is accompanied with wide-spread transcriptional dysregulation. To further evaluate the genome-wide transcriptional response to *Paip1* deficiency, we performed RNA sequencing (RNA-Seq) analysis on third instar larval wing discs dissected from the control and *tub-Gal4* driven *Paip1* depletion animals. Through this analysis, we identified 1353 differential regulated genes in *Paip1* depletion wing discs. Among them 850 were significantly upregulated, while 503 were downregulated (Fig. 6A). GO enrichment analysis found that upregulated genes were enriched in terms such as sulfur compound metabolic process, cellular modified amino acid metabolic process and glutathione metabolic process (Fig. 6Ba), while downregulated genes were linked to oxoacid metabolic process, carboxylic acid metabolic process and cuticle development (Fig. 6Bb). In addition, through manual annotation, we found that *Paip1* deficient cells displayed upregulation of genes associated with a set of signaling pathways such as the JNK signaling pathway, the JAK/STAT signaling pathway, the metabolic pathway, the oxidative stress pathway and DNA damage response pathway (Fig. 6C). Similar genome-wide transcriptional response was found in *Paip1*^*1*^ mutant (Supplementary Fig. S7A–D). The upregulation of a few selected genes were further verified using qPCR analysis (Supplementary Fig. S7E). As previous studies have shown that JNK activation is required for apoptotic cell death in response to various stress, we then asked whether *Paip1* depletion-induced apoptosis is mediated by JNK activation. The activation of JNK signaling was first confirmed by the upregulation of Mmp1, a known JNK target (Fig. 6D, E”, quantified in F). Furthermore, a dominant negative form of the *Drosophila* JNK gene *basket* (*UAS-bsk.DN*) was used to block JNK signaling in *Paip1* knockdown wing discs, and we found that reduction of JNK activity partially suppressed the apoptotic cell death, as revealed by the reduction of cleaved Dcp-1 signals (Fig. 6G–J”, quantified in K). These data indicate that activation of JNK is responsible for the apoptotic cell death upon loss of *Paip1*. In addition, we also found that increased Mmp1 expression in *Paip1* depletion wing discs was abolished by knockdown of *PERK*, suggesting that ISR functions upstream of JNK pathway in controlling apoptotic cell death (Supplementary Fig. S8A–D”, quantified in E). Together, our RNA-seq analysis identifies a molecular signature of response to *Paip1* deficiency and indicates that loss of *Paip1* induces activation of multiple signaling pathways.

**Fig. 7:**
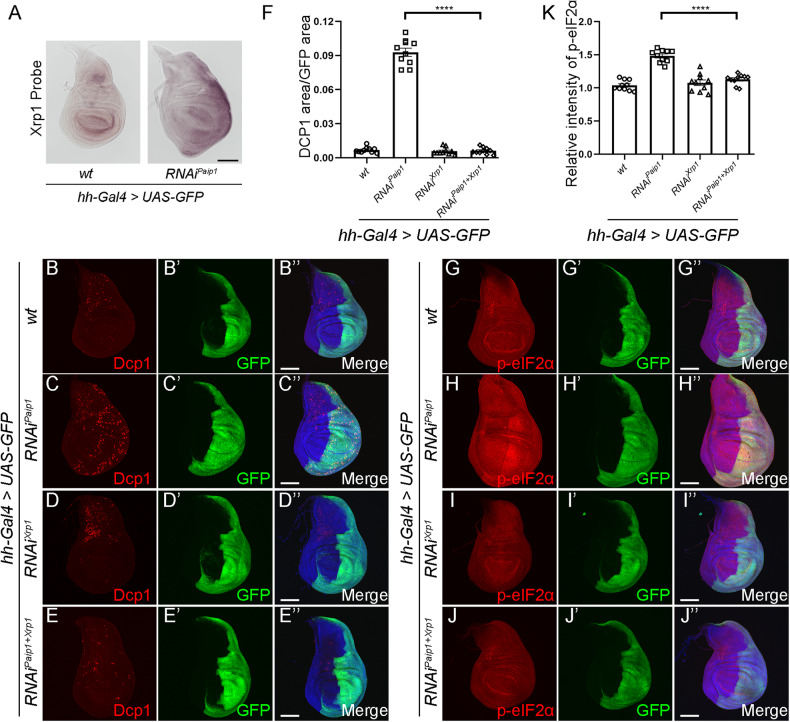
Knockdown of *Paip1* upregulates *Xrp1*, which contributes to apoptotic cell death and ISR activation. **A** Increased *Xrp1* expression in the posterior compartment of wing discs expressing *Paip1-RNAi*. **B**–**E**” *Xrp1* mediates cell death induced by *Paip1* knockdown. Wing imaginal discs from third instar larvae of control (**B**–**B**”), *Paip1-RNAi* (**C**–**C**”), *Xrp1-RNAi* (**D**–**D**”) and *Paip1-Xrp1 double RNAi* (**E**–**E**”) stained for Dcp1 (red), GFP (green) and DAPI (blue). **F** Statistical data of apoptotic cell death (Dcp1 area/GFP area) in (**E**–**H**”). **G**–**J**” *Xrp1* mediates phosphorylation of eIF2α in *Paip1* knockdown wing discs. Wing imaginal discs from third instar larvae of control (**G**–**G**”), *Paip1-RNAi* (**H**–**H**”), *Xrp1-RNAi* (**I**–**I**”) and *Paip1-Xrp1 double RNAi* (**J**–**J**”) stained for p-eIF2α (red), GFP (green) and DAPI (blue). Higher level of phosphorylated eIF2α upon *Paip1* knockdown (**H**–**H**”, labeled with GFP) was reduced by knocking down *Xrp1* simultaneously (**J**–**J**”). **K** Statistical data of p-eIF2α level in (**J**–**M**”). For (**A**), scale bars, 100 µm. For (**B**–**E**”) and (**G**–**J**”), scale bars, 100 µm. For (**F**) and (**K**), data are mean ± SEM. *n* = 10 discs per genotype. Statistical analysis was performed using a two-tailed unpaired *t*-test. ****P < 0.0001.

One of the upregulated genes, *Xrp1*, is of particular interest since it is also upregulated in ribosomal protein mutant clones and has an essential role in apoptotic cell death [24, 25, 40–42]. The elevated expression of *Xrp1* was verified in *Paip1* depletion cells using in situ hybridization (Fig. 7A). To determine whether *Xrp1* also participates in apoptotic cell death resulting from *Paip1* depletion, we performed rescue experiments by knocking down *Xrp1* and *Paip1* simultaneously. Our results show that *hh-Gal4* driven knockdown of *Xrp1* in a *Paip1* depletion background suppressed apoptotic cell death in the wing posterior compartment (Fig. 7B–E”, and quantified in F). Moreover, *Xrp1* knockdown also blocked the higher phosphorylation of eIF2α in *Paip1* depletion cells (Fig. 7G–J”, and quantified in K). It has been reported that Xrp1 expression results in JNK activation in *Rp/+* and *mahj* mutant cells [24, 27, 43], which prompted us to test the relationship between Xrp1 and JNK signaling in *Paip1* depletion wing discs. As expected, knockdown of *Xrp1* in a *Paip1* depletion background suppressed the elevated Mmp1 expression, suggesting elevated JNK activity is *Xrp1* dependent in *Paip1*-deficient cells (Supplementary Fig. S8F–I”, quantified in J). Together, these data suggest that loss of *Paip1* upregulates *Xrp1*, which contributes to apoptotic cell death and phosphorylation of eIF2α.

### Loss of Paip1 leads to an increase in Xrp1 translation efficiency

**Fig. 8:**
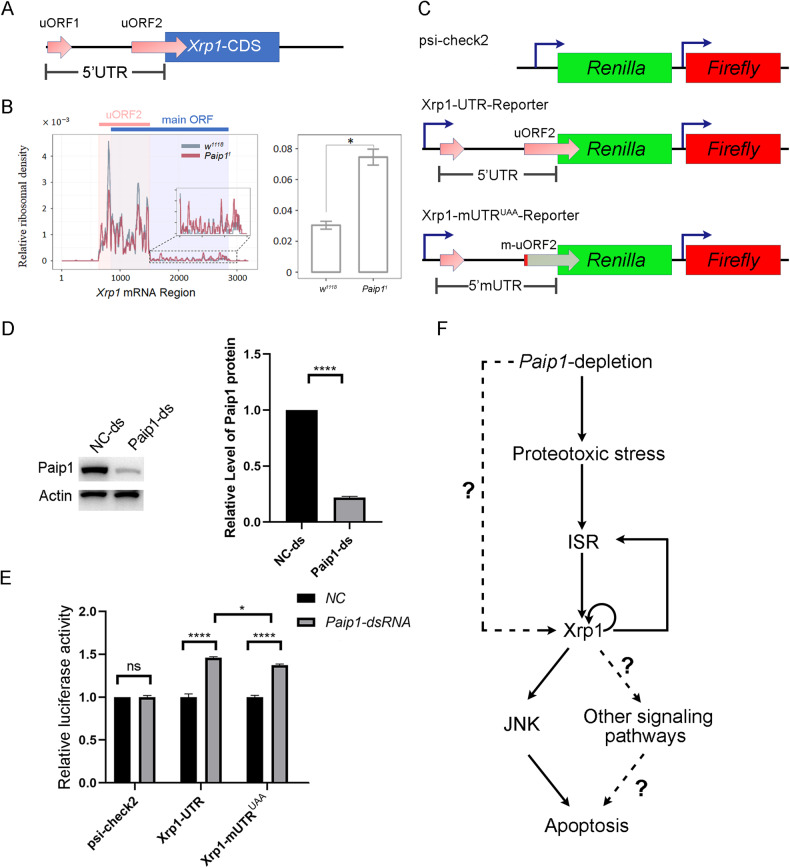
Loss of *Paip1* results in an increase in *Xrp1* translation mediated by its 5’UTR. **A** Schematic of structure of the 5’UTR of the *Drosophila Xrp1* mRNA. There are two predicted uORFs in the 5’UTR, and the uORF2 overlaps with the main CDS region in a different reading frame. **B** Relative ribosomal occupancy on *Xrp1* mRNA. **C** Schematic of luciferase-reporters for control, wild-type *Xrp1* 5’UTR and mutated *Xrp1* 5’UTR. **D** Western blot analysis of Paip1 dsRNA transfection efficiency. Western blot of control and *Paip1* knockdown cell lysate probed with anti-Paip1 and anti-Actin antibodies. Quantification was shown in the barplot. Data are mean ± SEM, *n* = 3. Statistical analysis was performed using a two-tailed unpaired *t*-test. ****P < 0.0001. **E** Effect of *Paip1-depletion* on the luciferase reporter activity (psi-check2 control plasmid, *Xrp1*-UTR-reporter, and *Xrp1*-mUTR^UAA^-reporter). Relative luciferase activity was measured by *Renilla* activity*/Firefly* activity and normalized to NC-psi-check2. Data are mean ± SEM, *n* = 3. Statistical analysis was performed using a two-tailed unpaired *t*-test. ****P < 0.0001; *P < 0.05; ns, *P* > 0.05. **F** Working model describing the roles of ISR and Xrp1 on apoptosis in *Paip1* depletion cells.

As Paip1 is a translational regulator, we next wanted to examine the relationship between *Paip1* deletion-mediated translation effect and *Xrp1* expression. It has been previously reported that *Xrp1* autoregulates its transcription through a positive autoregulatory loop [40]. In another study, it has been shown that *Xrp1* 5’UTR contains two putative upstream open reading frames (uORF, uORF1 and uORF2) and the uORF2 overlaps with the main ORF in a different reading frame, which indicates a possible translational regulation of *Xrp1* mediated by its 5’UTR (Fig. 8A) [41]. Thus, we speculated that loss of *Paip1* might increase *Xrp1* translation through its 5’UTR, leading to further upregulation of its transcription. To test this idea, we first carried out a Ribo-Seq analysis for RNA samples from wild-type and *Paip1*^*1*^ mutants (Supplementary Fig. S9). Our Ribo-seq data revealed that the ribosomal occupancy within the uORF2 of *Xrp1* 5’ UTR was reduced and that within *Xrp1* CDS was increased in *Paip1* mutant larvae as compared to the control (Fig. 8B). Thus, the effective translation efficiency of *Xrp1* is higher upon *Paip1* depletion. Moreover, we examined whether *Xrp1* 5’UTR and uORF2 are involved in the *Xrp1* translational regulation upon *Paip1* depletion. For this purpose, we cloned the 5’-UTR sequence of *Xrp1* into a luciferase reporter (upstream of *Renilla* luciferase) and examined the luciferase activity upon *Paip1* depletion in *Drosophila* S2 cells (Fig. 8C). This reporter enabled us to determine the effect of the 5’UTR on the translation of *Renilla* reporter coding sequence. The knockdown efficiency of *Paip1* by dsRNA was confirmed by western blot analysis (Fig. 8D). We found that *Paip1* depletion caused the upregulation of luciferase activity, indicating that *Xrp1* translational regulation is mediated by its 5’UTR (Fig. 8E). In addition, we also made a reporter with uORF2 start codon mutated to UAA within the 5’UTR of *Xrp1* and performed the luciferase reporter assay. Our results showed that uORF2^UAA^ mutated 5’UTR of *Xrp1* displayed a partial reduction of luciferase activity as compared to the wild-type 5’UTR of *Xrp1* in *Paip1* knockdown cells (Fig. 8E). Despite this reduction, the luciferase activity for uORF2^UAA^ mutated 5’UTR of *Xrp1* was still upregulated upon *Paip1* knockdown (Fig. 8E). These results together suggest that *Xrp1* 5’UTR based translational regulation is partially dependent on this start codon of uORF2. Moreover, it is also likely that other downstream effects of *Paip1* mediate this translational regulation. Thus, loss of *Paip1* can lead to an increase in *Xrp1* translation mediated by its 5’UTR and uORF2.

## Discussion

We have shown that *Paip1*, the *Drosophila* homolog of human *PAIP1*, is required for animal development and its deletion causes developmental delay and pupal lethality. It has been reported that mammalian PAIP1 controls the circularization of mRNA to ensure translation initiation [11]. In our experiments, loss of *Drosophila Paip1* results in a strong reduction of the polysomes and a strong decrease in ribosomal mRNA translation, documenting a conserved nature of the function of *PAIP1* in translation control. It has been previously reported that mutations in genes encoding ribosomal proteins reduces overall growth and causes developmental delay in *Drosophila* [44]. Mutations in the translation initiation factors *eIF4E* and *eIF4A* cause a dramatic larval growth arrest phenotype [45, 46]. Together, these findings support the notion that dysregulation of translation is associated with growth defects.

Tissue-specific knockdown of *Paip1* in the wing imaginal discs triggers apoptotic cell death and induces proteotoxic stress. Activation of the integrated stress response pathway can promote adaptive cell survival or initiate programmed cell death, depending on the context [34, 35]. Loss of *Paip1* in *Drosophila* wing imaginal discs causes increased phosphorylation of eIF2α. Our genetic data demonstrate an important role of PERK in the induction of eIF2α phosphorylation in response to *Paip1* depletion. We further show that block of PERK-mediated eIF2α activation restores the cell death phenotype induced by *Paip1* depletion. These findings point to the important role of ISR activation in mediating apoptotic cell death in *Paip1* deficient cells (Fig. 8F). Previous studies have shown that ribosomal protein-deficient cells show increased proteotoxic stress and activation of the ISR [27, 28, 47]. It is likely that reduction of ribosomal mRNA translation in *Paip1* depletion cells leads to the imbalance of ribosome protein subunits, contributing to ISR activation.

We find that depletion of *Paip1* causes a genome-wide transcriptional response and upregulates many genes, including *Xrp1*. More importantly, the apoptotic cell death upon *Paip1* depletion is rescued by knockdown of *Xrp1*. Since *Xrp1* knockdown also restores the phosphorylation of eIF2α in *Paip1* knockdown wing discs, the role for *Xrp1* in mediating cell death might act upstream in controlling the stress response following reduction of *Paip1* (Fig. 8F). Through Ribo-Seq analysis, we find that loss of *Paip1* increases the ribosome occupancy within the CDS of *Xrp1* while reducing the ribosome occupancy within the 5’ UTR of *Xrp1*. These findings indicate the effective translation efficiency of *Xrp1* is increased upon *Paip1* depletion. We have shown that the 5’ UTR of *Xrp1* mediates its translational regulation in *Paip1* depletion cells. Furthermore, mutation of the start codon within the uORF2 of *Xrp1* 5’UTR partially alters the effect of translational regulation upon *Paip1* knockdown, suggesting a uORF-based translational regulation for *Xrp1*. Consistent with this, previous studies have shown that increased eIF2α phosphorylation results in efficient translation reinitiation at downstream AUGs in certain mRNAs with uORFs [48–51]. In addition, it is also likely that other downstream events of eIF2α phosphorylation contributes to *Xrp1* expression. Since Paip1 is a translational regulator, it is possible that there is a direct role of Paip1 in regulating *Xrp1* translation in vivo. The detailed molecular mechanism for *Xrp1* translational regulation warrants further investigation. In summary, our findings provide a novel insight in the role of ISR activation and *Xrp1* in cell death induced by translation impairment.

## Materials and methods

### Fly stocks and genetics

The following fly stocks were used: *w*^*1118*^, *Paip1*^1^, *Paip1-20K*, *UAS-Paip1-RNAi* (VDRC, v26916), *UAS-Xrp1-RNAi* (THFC, THU0591), *UAS-PERK-RNAi* (BDSC, 35162), *UAS-GCN2-RNAi* (BDSC, 67215), *UAS-bsk.DN* (BDSC, 6409), *UAS-crc-RNAi* (THFC, THU5856) *Tubulin-Gal4*, *hh-Gal4, UAS-lacZ, UAS-p35*. All the stocks and crosses were reared at 25 °C.

*Paip1-20K* transgenic line was generated using CH322-46O04 construct. CH322-46O04 construct covers 20k bp sequence of the *Drosophila* genome, including full length of Paip1. CH322-46O04 construct was injected into the attP2 landing site of UAS-phi2b2a; VK5 (75B1).

### Mutant generation

The *Paip1*^*1*^ mutant allele was generated through CRISPR-Cas9 system. The Paip1 target region was located between site 17018815 and site 17018833 on chromosome 2R (GGAGTGGCCATGCCGGGAC), and the synthesized gRNA was injected to vasa-Cas9 fly embryos (BL51323). Injected flies were crossed with flies carrying the balancer chromosomes. Mutations were determined by PCR screening and sequencing.

### Immunofluorescence staining and microscopy

Imaginal discs were dissected in cold PBS and fixed for 20 min in 4% paraformaldehyde in PBS. Samples were blocked for 1 h in 3% BSA in PBS and then incubated with primary antibodies overnight at 4 °C. After three times of PBT washes, samples were incubated with secondary antibodies for 2 h. DAPI was added for the last 20 min. Samples were mounted in Vectorshield. Images were obtained using an Olympus FV1000 confocal microscope.

The following primary antibodies were used: rabbit anti-Paip1(1:1000), rabbit anti- p-eIF2α (1:100, CST, 3398), rabbit anti- Dcp1(1:100, CST, 9578), mouse anti-Ubiquitin (1:400, CST, 3936), rabbit anti-Ref(2)P (1:1000, Abcam, ab178440), chicken anti-GFP (1:2000, Invitrogen), mouse anti Mmp1(1:20, DSHB, 3A6B4).

Anti-Paip1 antibody was raised in rabbit against a GST–Paip1 fusion protein. A cDNA fragment of *Paip1*, corresponding to the C-terminal region, was cloned into the pGEX-4T1 vector. After the immunogen purification and rabbit injection, anti-sera were collected from the 4th boost and affinity purified.

For ProteoStat staining, we used the commercial PROTEOSTAT^®^ Aggresome detection kit (Enzo Life, ENZ-51035). Briefly, imaginal discs were dissected in PBS and fixed in 4% formaldehyde diluted in 1X Assay Buffer for 30 min at room temperature, then washed twice with PBS. The samples were subsequently permeabilized in permeabilizing solution (0.5% Triton X-100, 3 mM EDTA, pH 8.0, diluted by 1X Assay Buffer) on ice for 30 min, and washed twice by PBS. Removed excess buffer and added Detection buffer (Detection Reagent, 1:500 in 1X Assay buffer), protect samples from light and incubate for 30 min at room temperature. Washed samples with PBS, then removed excess buffer and mounted samples by Antifade Mounting Medium with DAPI (Beyotime, P0131). Images were obtained using an Olympus FV1000 confocal microscope.

### Western blotting

For western blotting analysis, whole larvae or wing discs were lysed in RIPA lysis buffer [50 mM Tris-HCl pH 8.0, 150 mM NaCl, 1% (v/v) SDS, 0.5% (w/v) sodium deoxycholate, complete protease inhibitor cocktail and PhosStop phosphatase inhibitor cocktail. The following antibodies were used: HRP-conjugated β-Actin Rabbit mAb (1:5000, ABclonal, AC028), HRP-conjugated β-Tubulin Mouse mAb (1:5000, ABclonal, AC030), rabbit anti-Paip1 (1:1000), mouse anti-RPS6 (1:1000, CST, 2317) and rabbit anti-RPL40 (1:1000, abcam, ab109227).

### Image acquisition, processing, and Statistics

Images of whole pupae and adults were acquired using Nikon SMZ18 microscope with 3× objective. Images of in situ hybridized discs were acquired using Nikon eclipse 80i microscope with 20× objective.

Confocal images were acquired using Olympus FV1000 confocal microscope with 20× and 40× objectives. All wing imaginal discs were imaged as z-stacks with each section corresponding to 1 μm (20×) or 0.5 μm (40×). Images were subsequently analyzed by using image-J and processed by Adobe Photoshop CC. For Dcp1 and ProteoStat quantifications, DCP1 positive cells were counted in the region specified in each experiment (posterior area marked by GFP, or whole disc region by DAPI, as described in the figure legend). All counts were normalized to their respective area measured by image-J. 10 discs were quantified for each genotype. Quantifications of p-eIF2α, ubiquitin, Ref(2)P and Mmp1 were performed in image-J with the mean gray intensity measurement, and signals in GFP positive region were normalized by GFP negative region.

Statistical tests used for each experiment are shown in figure legend. GraphPad Prism 8 was used to perform statistical tests. Two-tailed unpaired t-test was used for Dcp1, ProteoStat, p-eIF2α, ubiquitin, Ref(2)P, and Mmp1 quantifications.

### Polysome profiling and polysome sequencing

About 100 third instar larvae were collected and grinded with liquid nitrogen using a mortar and pestle. Then lysed samples in lysis buffer [10 mM Tris-HCl (pH 7.4), 5 mM MgCl_2_, 100 mM KCl, 1% Triton X-100, 2 mM DTT, 100 µg/ml CHX, 500U/ml RNasin Plus (Promega), cOmplete EDTA-free protease inhibitor] by grinding them 20 times with a Dounce tissue grinder on ice. The lysate was centrifuged for 10 min at 15,000 rpm and transferred supernatant into a new tube. The concentration of RNA in each sample was measured using Nanodrop2000. We prepared gradients using a Gradient Master (Biocomp, 10% sucrose solution: 10% sucrose (w/v), 10 mM Tris-HCl (pH 7.4), 5 mM MgCl_2_, 100 mM KCl, 2 mM DTT; 50% sucrose solution: 50% sucrose (w/v), 10 mM Tris-HCl (pH 7.4), 5 mM MgCl_2_, 100 mM KCl, 2 mM DTT). Total RNAs were extracted from 10% lysates with TRIzol reagents (Ambion) and followed by RNA-seq, then loaded lysates on the gradients. Samples were centrifuged in an SW-41 Ti rotor at 36,000 rpm at 4 °C for 2.5 h, and using a Piston Gradient Fractionator (Biocomp) according to the manufacturer’s instructions to collect fractions.

For polysome sequencing, monosome and polysome fractions were collected separately. Before RNA extraction, Equal amounts of ERCC RNA Spike-in mix (Thermo, 4456740) were added as RNA input control (Ref). We extracted RNA in each fraction through phenol-chloroform extraction. The construction of the library was completed with VAHTS Universal V8 RNA-seq Library Prep Kit for Illumina (Vazyme, NR605). Libraries were sequenced on the Novaseq 6000 (Nanjing jiangbei New Area biopharmaceutical Public service Platform). We applied Hisat2 to align the sequencing reads, the subsequent mapped reads were quantified by featureCounts. The genome reference of *Drosophila* used here is dmel-6.39 (NCBI). We achieved ERCC RNA spike-in alignments and normalization according to the manufacturer’s instructions, then divided polysome by monosome to obtain translation efficiency (TE). Identification of differential expressed genes and GO enrichment analysis were using R packages edgeR, topGO, and clusterProfiler.

### Ribo-seq

Ribo-seq was performed as described [52]. Briefly, the same protocol for Polysome profiling was used for larvae preparation. Total RNAs were extracted from 10% lysates with TRIzol reagents (Ambion) and followed by RNA-seq as input. Then 15 mM CaCl_2_ was added into lysates, and samples were treated with MNase (3U/μg) for 45 min at 25 °C. The digestion was quenched by 6.25 mM EGTA. After sucrose gradient centrifugation, the monosome fractions were collected and RNAs were extracted through phenol-chloroform extraction. By denaturing polyacrylamide gel, we selected 28-32nt ribosome-protected RNA fragments for library construction. Throughout the protocol, SuperaseIn was applied. To remove rRNA, RNAs were treated with RiboMinus Kit (Invitrogen, K155001). The remain RNAs were sequentially subjected to adapter ligation, reverse transcription, secondly rRNA-depletion and PCR amplification. The adapters, primers, and rRNA-depletion oligos were used as described in a previous study [52]. Libraries were sequenced on the Novaseq 6000 (Nanjing jiangbei New Area biopharmaceutical Public service Platform). Reads alignments and data processing were performed by Hisat2 (v2.2.1), featureCounts (v2.0.1) and R studio (v4.1.0). Each genotype has two replicates.

### Ribosome occupancy analysis

All RPFs were aligned directly to transcripts using Bowtie (v1.0.0). We mapped RFPs for specific transcripts, and obtained the accumulated reads density per base along transcript, then normalized by total number of stacked counts at all positions, referred to as relative ribosome occupancy (i.e., sum of all position densities is 1). After annotation of RNA regions, the proportions on different structures were calculated.

### RNA sequencing

Fifty imaginal discs per sample were dissected and lysed in TRIzol reagents (Ambion). Then extracting total mRNA according to the manufacturer’s instructions. The construction of the library was completed with VAHTS Universal V8 RNA-seq Library Prep Kit for Illumina (Vazyme, NR605). Libraries were sequenced on the Novaseq 6000 (Nanjing jiangbei New Area biopharmaceutical Public service Platform). At least 20,000,000 clean reads were obtained from every sample and at least 90% of reads were mapped from every sample. Reads alignments and data processing were performed by Hisat2 (v2.2.1), featureCounts (v2.0.1) and R studio (v4.1.0). Each genotype has 2 replicates.

### In situ hybridization

In situ hybridization was performed as described [53]. Briefly, digoxigenin-labeled Xrp1 probe was synthesized following standard methods (Primers: Xrp1probe F-CGGGATGTGAGTGGAGCAAT, Xrp1probe R-ATAGGGGTCCTCTGAGCTGG, and T7 promoter sequence was added to reverse primer). Dissect 3rd instar larval wing discs from indicated genotypes and washed with PBS. Then fixed with 4% paraformaldehyde. Samples were washed three times in PBT and permeabilized in xylenes. Before hybridization, discs were incubated in prehybridization solution. Then hybridized with digoxigenin-labeled RNA probe in a 65 °C incubator rotating overnight. The next day, wing discs were incubated with AP-conjugated digoxigenin antibody (Roche) and color was developed with NBT/BCIP solution. After staining, discs were mounted with glycerol for imaging.

### QPCR

Total RNAs were extracted by TRIzol reagent from 50 imaginal discs. RT was performed by SuperScript™ IV Reverse Transcriptase (Invitrogen, 18090200). Real-time PCR was performed using Bioen LineGene9600 with 2X Universal SYBR Green Fast qPCR Mix (ABclonal, RK21203). Three independent RT experiment were performed and data were normalized against *actin* mRNA level. The primers used are shown in Table S1.

### Plasmid construction

*Xrp1*-5’UTR fragment was obtained through RT-PCR (Primers: *Xrp1*-5’UTRF- AGTAATCCATGTACAAAATAACCAAC, *Xrp1*-5’UTRR -CAATATTATATCTCTGGGAT) and subcloned into the psiCHECK2 vector (Promega, C8021) upstream of *Renilla*-luciferase. Briefly, the psiCHECK2 vector was first linearized by Nhe I (NEB, R3131V). The ClonExpress II One Step Cloning Kit (Vazyme, C112) was used to ligate *Xrp1*-5’UTR fragment and linearized vector. The uORF2^UAA^ mutated construct was generated using the QuickMutation™ Site-directed Gene Mutagenesis Kit (Beyotime, D0206S). The sequence of mutated DNA was verified by DNA sequencing.

### Double-stranded RNA synthesis

Paip1 dsRNA sequence was designed using the dsCheck online software (http://dsCheck.RNAi.jp/). Negative control (NC) dsRNA sequence was designed against GFP. The T7 promoter sequence (TAATACGACTCACTATAGG) was added to the 5′terminus of Paip1 primers for dsRNA generation. The following primers were used (5’-3’): Paip1ds-F: GAGCGAGAGCAACGAAAATGTTCGC, Paip1ds-R: GCCCAAGCTATATCCTGCCAACGAC, NCds-F: GGCACAAATTTTCTGTCCGT, NCds-R: TCTTTTGTTTGTCTGCCGTG. dsRNA was synthesized using the MEGAscript RNAi Kit (Ambion, AM1626) and transfected into S2 cells following the standard protocol.

### S2 cell culture and transfection

*Drosophila* Schneider 2 (S2) cells were grown at 25 °C in Schneider’s *Drosophila* medium (Gibco, 21720024) supplemented with 10% fetal bovine serum (Sigma, F8318). For transfection, 12-well tissue culture plates were seeded with 6 × 10^5^ S2 cells per well. Transfections were performed with jetPRIME reagent (Polyplus, 101000046) according to the manufacturer’s instructions.

### Luciferase assays

Luciferase activity was measured 48 h after transfection using the Dual-Luciferase® Reporter Assay System (Promega, E1960) according to the manufacturer’s instructions. Briefly, cells were lysed in 100 μl PLB per well, then lysates were centrifuged at 13,000 × *g* for 5 min to remove cell debris. *Firefly* and *Renilla* luciferase activity were measured on 20 μl lysate. *Firefly* luciferase functions as a transfection efficiency control.

### Supplementary information

SUPPLEMENTAL MATERIAL

aj-checklist

Original Data File

## Data Availability

Sequence data is available in the NCBI SRA (Sequence Read Archive) database under the BioProject accession number PRJNA935933 (Polysome profiling of *Paip1*^*1*^ mutant larvae), PRJNA935686 (RNA-seq of *Paip1* knockdown wing discs), PRJNA978514 (RNA-seq of *Paip1*^*1*^ mutant wing discs), PRJNA978515 (Ribo-seq of *Paip1*^*1*^ mutant larvae) and PRJNA979342 (RNA-Seq of *Paip1*^*1*^ mutant larvae as input for Ribo-seq).
