# Supplementary material for: Loss of Paip1 causes translation reduction and induces apoptotic cell death through ISR activation and Xrp1
Source: Cell Death Discov. 2023 Aug 5;9:288. doi: 10.1038/s41420-023-01587-8 (PMC10404277; doi:10.1038/s41420-023-01587-8)
Supplement: Supplementary file 1 — SUPPLEMENTAL MATERIAL [file 41420_2023_1587_MOESM1_ESM.pdf]

Supplementary data

Supplementary Figure S1

Supplementary Figure S2

Supplementary Figure S3

Supplementary Figure S4

Supplementary Figure S5

Supplementary Figure S6

Supplementary Figure S7

Supplementary Figure S8

Supplementary Figure S9

Supplementary Table S1

Supplementary Table S2

Figure S1

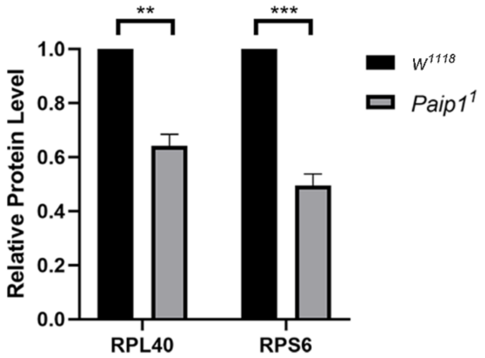

Figure S1 Quantification of western blot data in Figure 1H.

Data are mean ± SEM, n = 3. Statistical analysis was performed using a two-tailed unpaired t-test. **P<0.01, *** P<0.001.

Figure S2

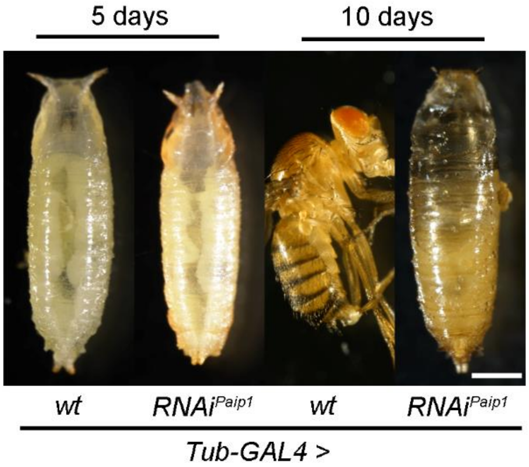

Figure S2 Knockdown of *Paip1* by *tubulin-Gal4* causes pupal lethality.

Images of pupae and adult flies with the indicated genotype. Pupae and adult flies of control and *Tub>Paip1-RNAi* were collected 5 days after egg hatching and 10 days after egg hatching. Scale bars, 500 μm.

Figure S3

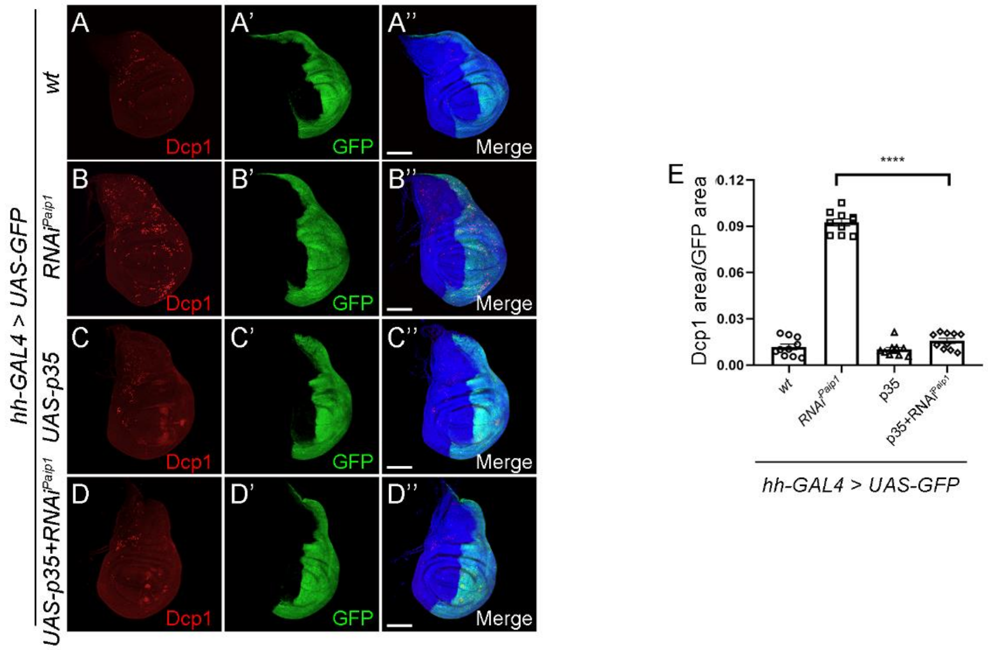

Figure S3 Overexpression of *p35* inhibits apoptosis induced by *Paip1* depletion.

(A-D") Wing imaginal discs from third instar larvae of control (A-A"), *Paip1-RNAi* (B-B"), *UAS-p35* (C-C") and *UAS-p35+Paip1-RNAi* (D-D") stained for Dcp1 (red), GFP (green) and DAPI (blue).

(E) Statistical data of apoptotic cell death (Dcp1 area/GFP area) in A-D".

For A-D", scale bars, 100 µm. For E, data are mean ± SEM. n = 10 discs per genotype. Statistical analysis was performed using a two-tailed unpaired t-test. **** P<0.0001.

Figure S4

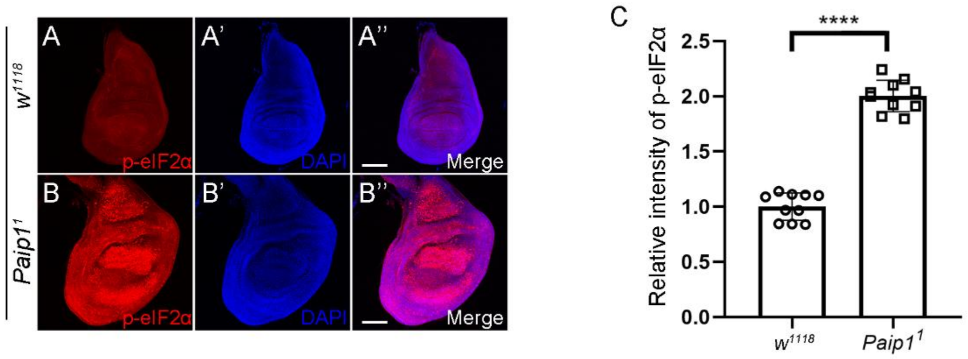

Figure S4 Elevated eIF2α phosphorylation level in *Paip1¹* mutant wing discs.

(A-B") Wing imaginal discs from third instar larvae of w^{1118} (A-A") and $Paip1^{1}$ (B-B") stained for p-eIF2α (red) and DAPI (blue).

(C) Statistical data of p-eIF2α level in A-B".

For A-B", scale bars, 100 µm. For C, data are mean ± SEM. n = 10 discs per genotype. Statistical analysis was performed using a two-tailed unpaired t-test. **** P<0.0001.

Figure S5

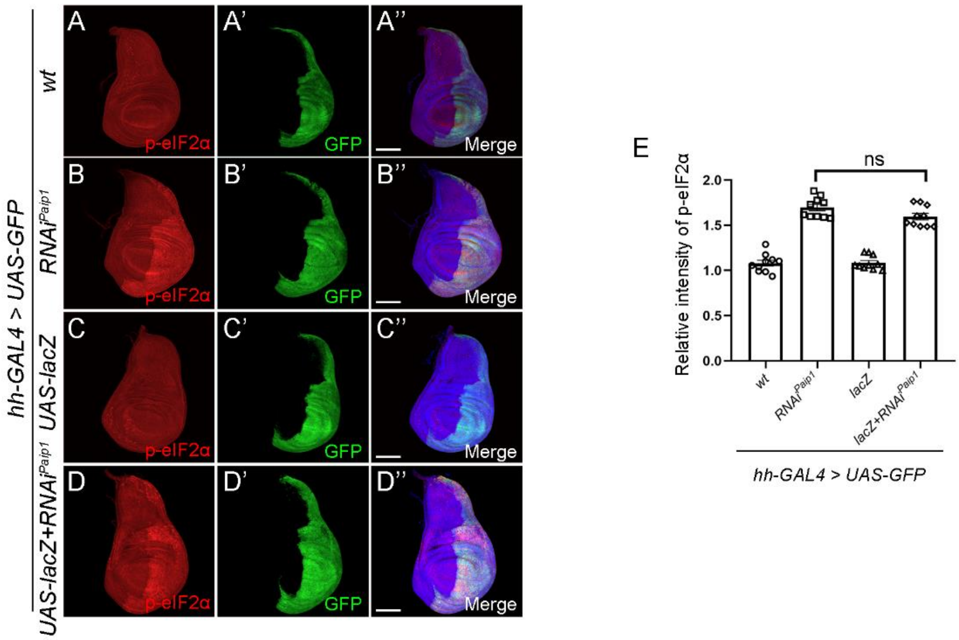

Figure S5 Overexpression of *lacZ* does not alter eIF2α phosphorylation level in *Paip1* depletion wing discs.

(A-D”) Wing imaginal discs from third instar larvae of control (A-A”), *Paip1-RNAi* (B-B”), *UAS-lacZ* (C-C”) and *UAS-lacZ+Paip1-RNAi* (D-D”) stained for p-eIF2α(red), GFP (green) and DAPI (blue).

(E) Statistical data of p-eIF2α level in A-D”.

For A-D”, scale bars, 100 μm. For E, data are mean ± SEM. n = 10 discs per genotype. Statistical analysis was performed using a two-tailed unpaired t-test. ns, $P>0.05$.

Figure S6

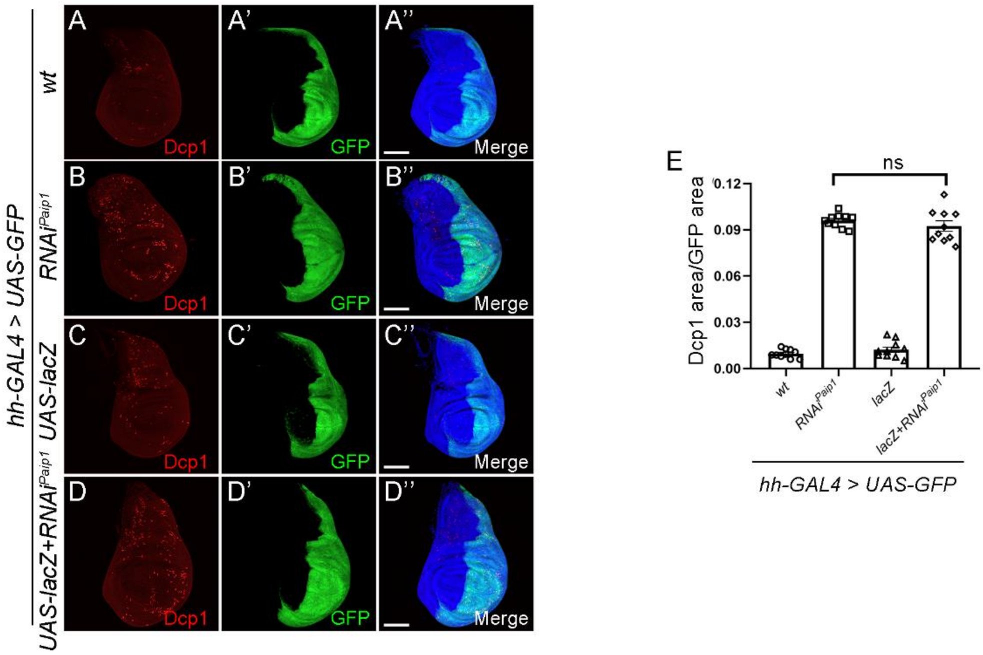

Figure S6 Overexpression of *lacZ* does not alter apoptotic cell death level in *Paip1* depletion wing discs.

(A-D") Wing imaginal discs from third instar larvae of control (A-A"), *Paip1-RNAi* (B-B"), *UAS-lacZ* (C-C") and *UAS-lacZ+Paip1-RNAi* (D-D") stained for Dcp1 (red), GFP (green) and DAPI (blue).

(E) Statistical data of apoptotic cell death (Dcp1 area/GFP area) in A-D".

For A-D", scale bars, 100 μm. For E, data are mean ± SEM. n = 10 discs per genotype. Statistical analysis was performed using a two-tailed unpaired t-test. ns, P>0.05.

Figure S7

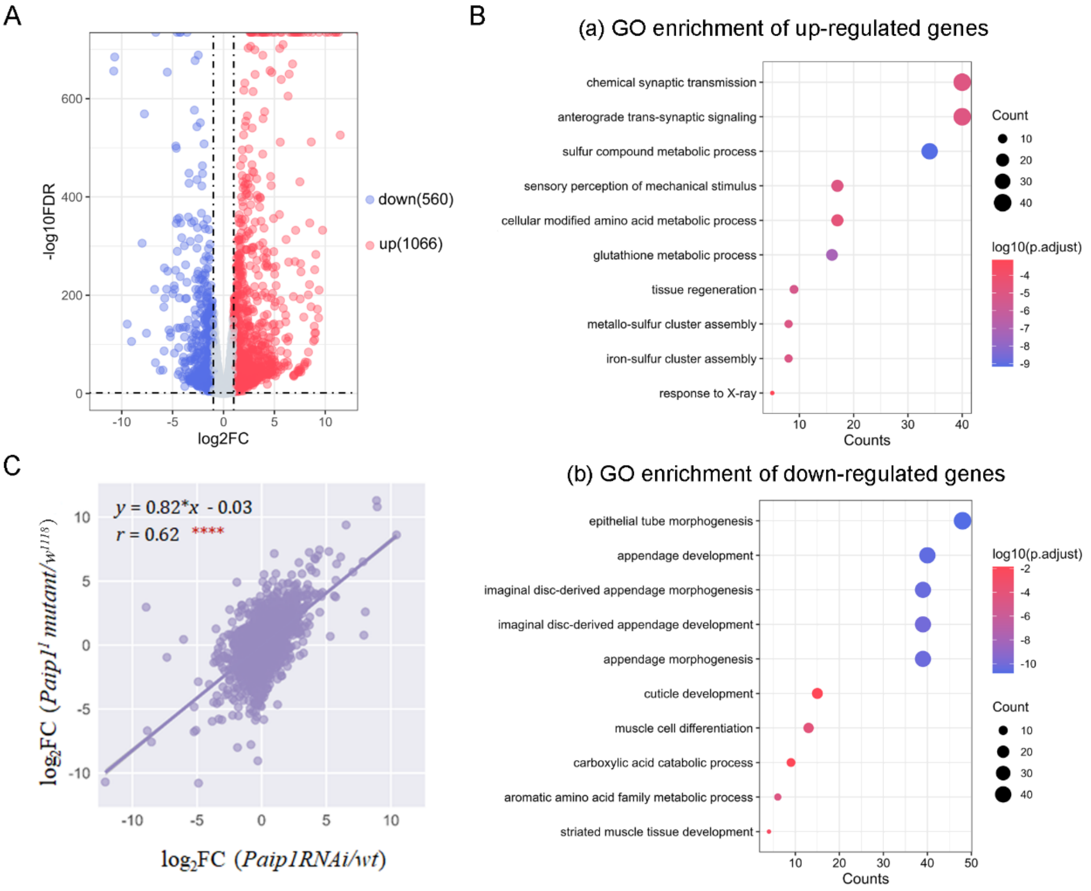

D

Pathway	Gene Name	log2FC(*Tub>Paip1 RNAi / Tub>wt*)	log2FC(*Paip1 mutant / w1118*)
JNK pathway	Gadd45	3.35	1.47
	Mmp1	1.63	3.29
	puc	0.75	1.37
JAK/STAT pathway	upd1	0.98	3.79
	upd2	4.48	7.44
	upd3	3.12	5.57
	Socs36E	1.01	2.34
Metabolic regulator	Ldh	7.84	6.51
	Pvf1	2.00	2.35
	GstD1	1.18	0.91
	GstD5	5.93	4.93
Oxidative stress	AOX1	4.15	6.70
	Arc2	4.35	7.00
DNA damage response	Xrp1	1.67	1.49
	Irbp18	2.09	1.86
ER stress	PEK	1.11	0.99

E

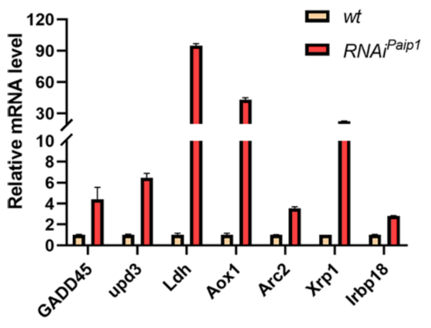

Figure S7 Genome-wide transcriptional response in *Paip1¹* mutant wing discs by RNA-seq analysis.

(A) Volcano plots of differentially expressed genes between control (*w¹¹¹⁸*) and *Paip1¹* wing discs. The numbers of differentially expressed gene are indicated (FDR<0.05 and |log2FC|>1), in which blue and red denote down- and up- regulated genes, respectively. Each genotype includes two replicates.

(B) GO enrichment analysis for up-regulated (a) and down-regulated genes (b). The significant level is 0.05.

(C) Correlation analysis of RNA-seq data from *Tub>Paip1-RNAi* and *Paip1¹ mutant*. Scatter plot showed relationship between log_2FC (*Paip1 mutant / w¹¹¹⁸*) and log_2FC (*Tub>Paip1-RNAi / Tub>wt*). Pearson correlation coefficient (r)= 0.62, ****P<0.0001.

(D) Fold changes of representative genes for selected signaling pathway through manual annotation in two groups.

(E)Q-PCR analysis of the indicated genes using mRNA from control and *Paip1* knockdown wing discs. Each gene has three replicates.

Figure S8

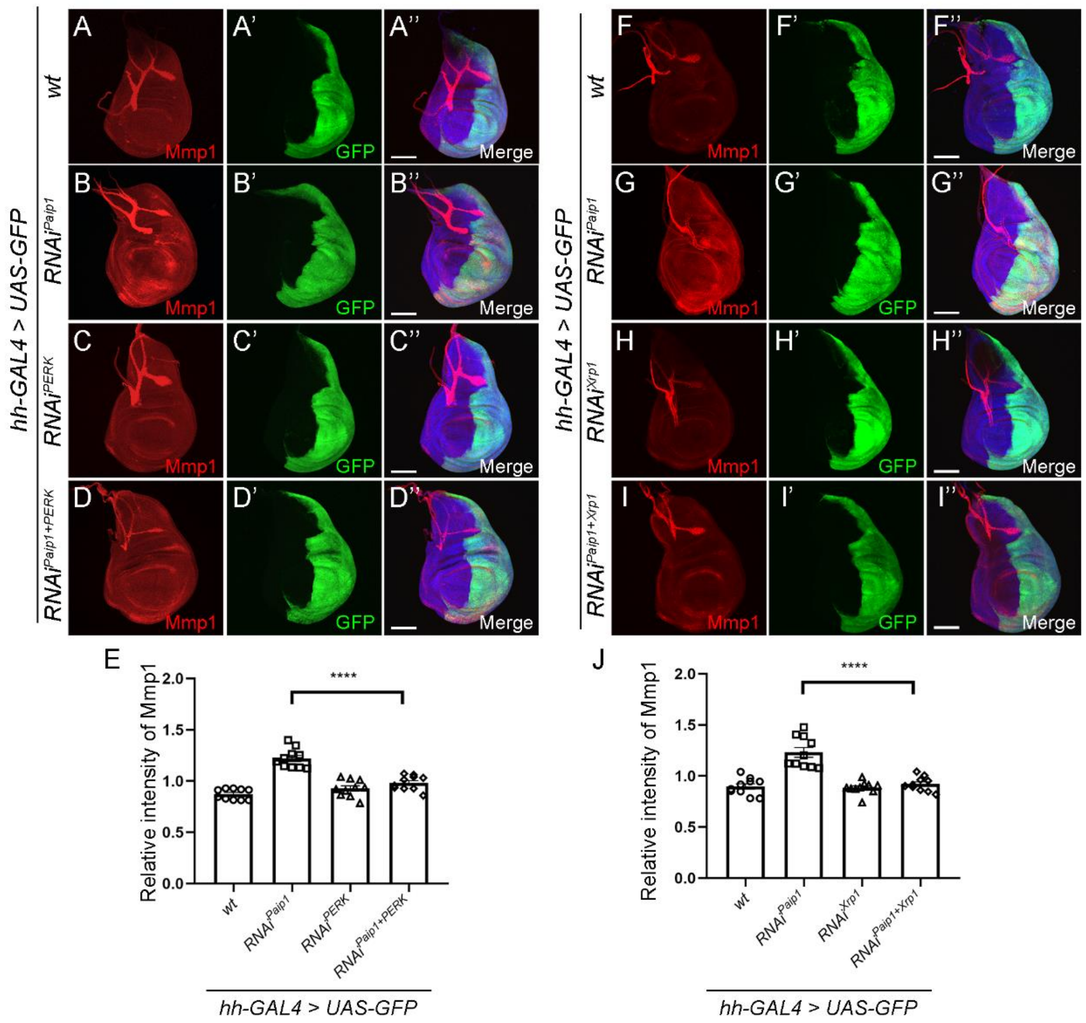

Figure S8 Knockdown of *PERK* or *Xrp1* reduces JNK signal.

(A-D") Wing imaginal discs from third instar larvae of control (A-A"), *Paip1-RNAi* (B-B"), *PERK-RNAi* (C-C") and *Paip1-RNAi*+ *PERK-RNAi* (D-D") stained for Mmp1(red), GFP (green) and DAPI (blue).

(E) Statistical data of Mmp1 level in A-D".

(F-I") Wing imaginal discs from third instar larvae of control (F-F"), *Paip1-RNAi* (G-G"), *Xrp1-RNAi* (H-H") and *Paip1-RNAi*+ *Xrp1-RNAi* (I-I") stained for Mmp1(red), GFP (green) and DAPI (blue).

(J) Statistical data of Mmp1 level in F-I".

Figure S9

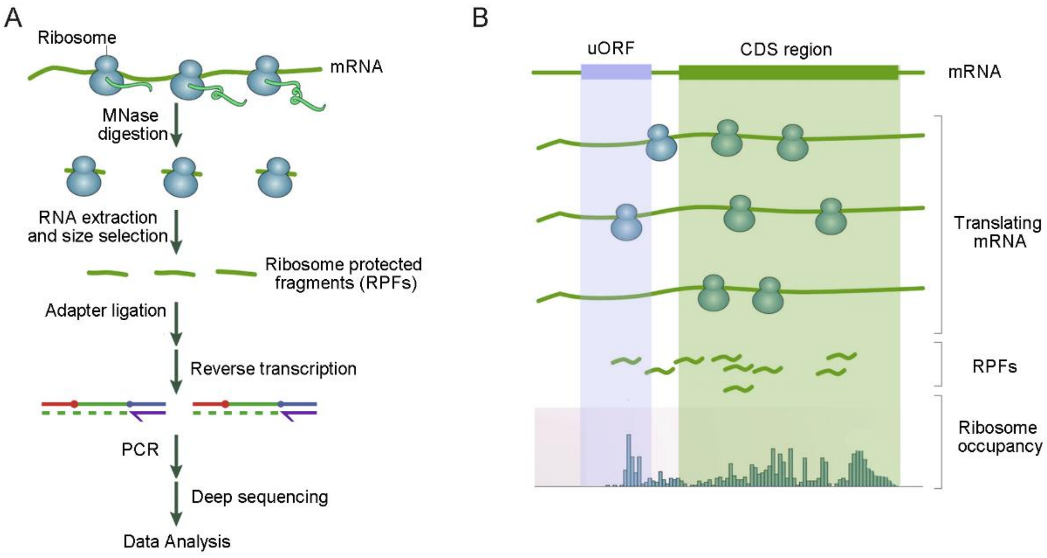

Figure S9 Schematic of library construction and data analysis for Ribo-seq.

(A) Library construction of Ribo-seq. Polysomes were digested by MNase, and ribosome protected fragments (RPFs) were obtained by extracting RNA from the digest and size selection. These RPFs were converted into a DNA library through adapter ligation, reverse transcription and PCR amplification.

(B) Ribosome occupancy data were obtained by mapping RPFs to the corresponding transcripts. After annotation of the RNA regions, the proportions on different structures were calculated.

Table S1 The lethality of *Paip1*^{1} at every developmental stage

Stage	***w***^{1118}			***Paip1***^{1}		
	**Replicate 1**	**Replicate 2**	**Replicate 3**	**Replicate 1**	**Replicate 2**	**Replicate 3**
Egg	100	100	100	100	100	100
1st Larva	98	98	99	95	92	96
3rd Larva	93	92	92	89	85	90
Pupa	92	89	90	88	83	87
Adult	92	89	90	0	0	0

Table S2 List of Primers used for Q-PCR analysis

**Gene**		**Primer Sequence(5'-3')**
Actin 42A	F	GCGTCGGTCAATTCAATCT
	R	AAGCTGCAACCTCTTCGTCA
GADD45	F	TGGAGGCCTTTTGCTACGAG
	R	ATTCCCTCGGGAACTCTCCA
upd3	F	GACAAAGTCGCCTGATTCGC
	R	GCGGTCAGCTGTCGTCATTT

ldh	F	CGAGCTGAACCCCATTCTGG
	R	ATGCCATGTTCGCCCAAAAC
AOX1	F	CGTGGAATCAAACGAGGCTG
	R	GGCAGACAGGTTGACTTCGT
Arc2	F	CGAAGCAGTCCTACGACGAA
	R	ATCTGCCCAATAAAGGCGCT
Xrp1	F	TGTGTGTGCAACATTCGCAG
	R	ACAAGTTCCCCTTAAACCTCCA
Irbp18	F	TGTGTTTTCTTCTGCATTTCCGT
	R	GTGCGCTGAACAGCCTCATT
