# Supplementary figures and images for: Loss of Paip1 causes translation reduction and induces apoptotic cell death through ISR activation and Xrp1
Source: Cell Death Discov. 2023 Aug 5;9:288. doi: 10.1038/s41420-023-01587-8 (PMC10404277; doi:10.1038/s41420-023-01587-8)

Figure 1C

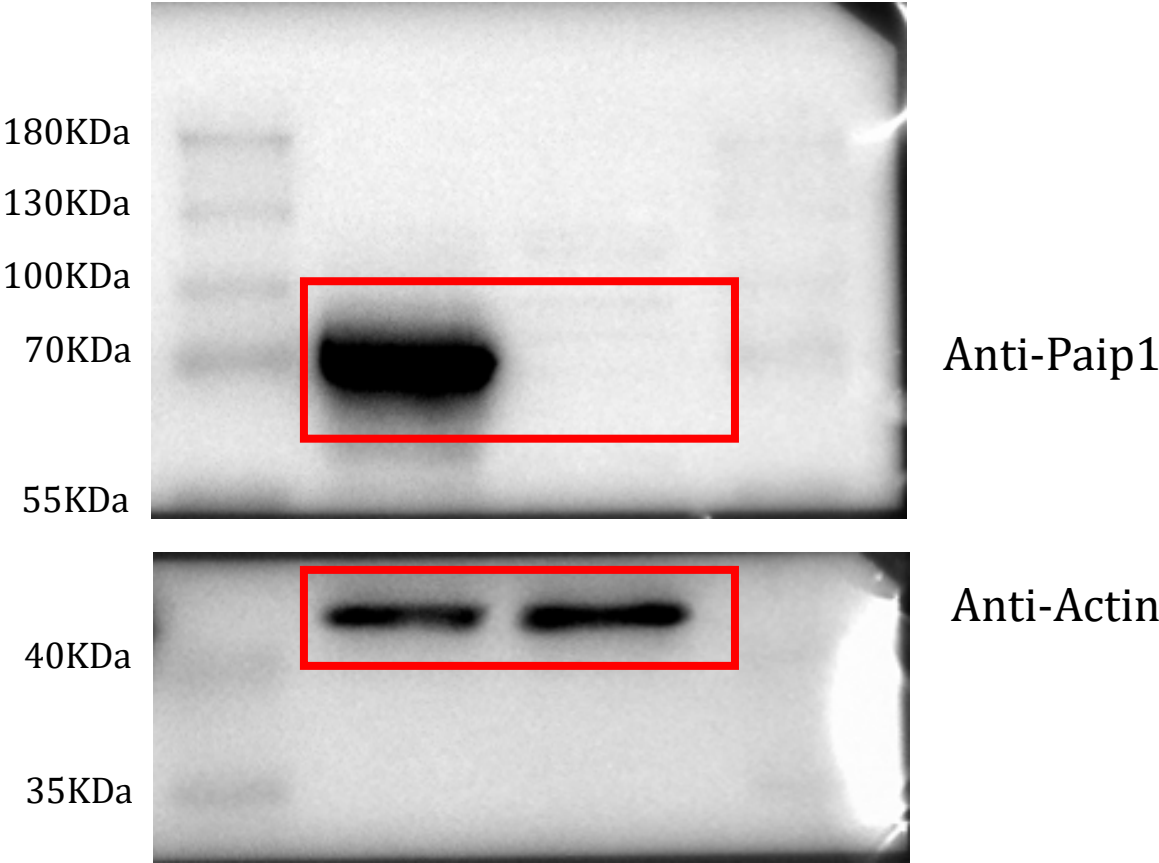

Figure 1H

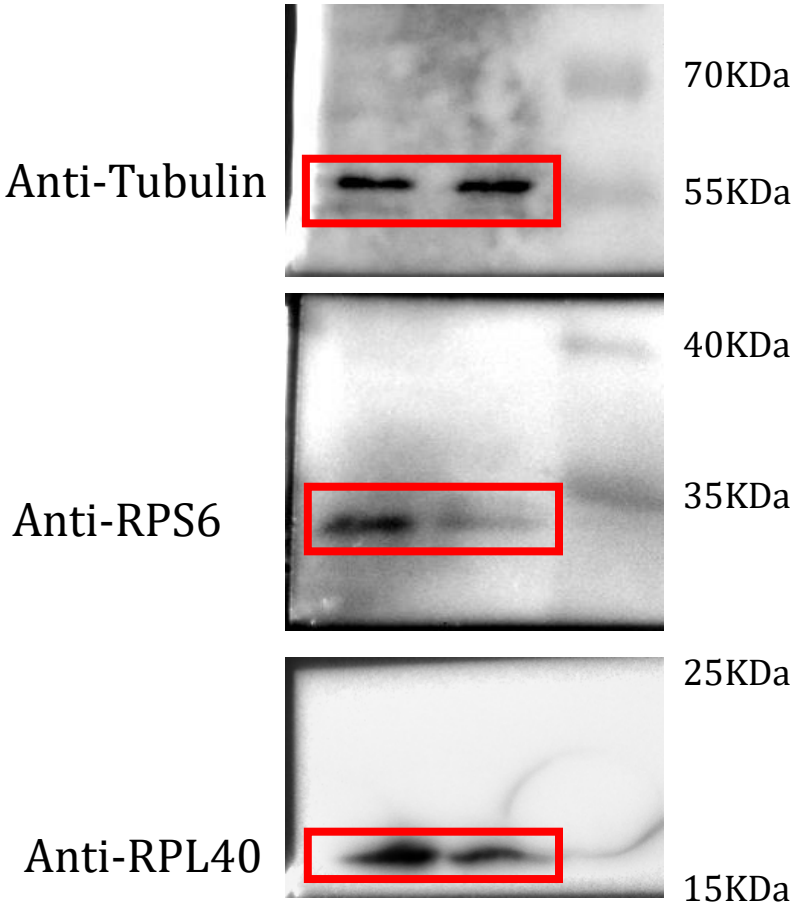

Figure 8D

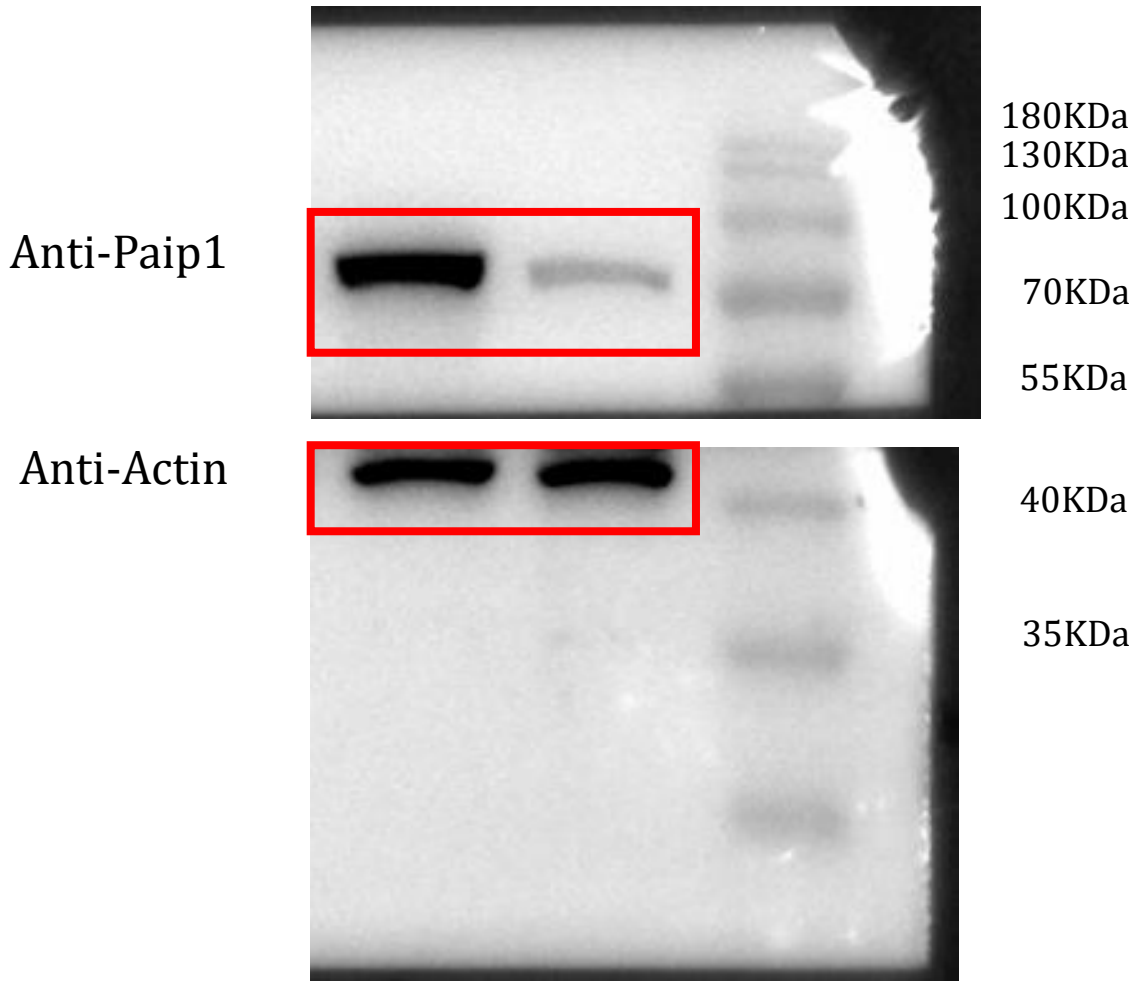

Supplement: Supplementary file 3 — Original Data File [file 41420_2023_1587_MOESM3_ESM.pdf]
